# Senescent mouse cells fail to overtly regulate the HIRA histone chaperone and do not form robust Senescence Associated Heterochromatin Foci

**DOI:** 10.1186/1747-1028-5-16

**Published:** 2010-06-22

**Authors:** Alyssa L Kennedy, Tony McBryan, Greg H Enders, F Brad Johnson, Rugang Zhang, Peter D Adams

**Affiliations:** 1 Graduate Program in Molecular and Cellular Biology and Genetics, Drexel University College of Medicine, Philadelphia, USA; 2 Fox Chase Cancer Center, Philadelphia, USA; 3 CR-UK Beatson Labs, Glasgow University, Glasgow, UK; 4 University of Pennsylvania, Philadelphia, USA

## Abstract

**Background:**

Cellular senescence is a permanent growth arrest that occurs in response to cellular stressors, such as telomere shortening or activation of oncogenes. Although the process of senescence growth arrest is somewhat conserved between mouse and human cells, there are some critical differences in the molecular pathways of senescence between these two species. Recent studies in human fibroblasts have defined a cell signaling pathway that is initiated by repression of a specific Wnt ligand, Wnt2. This, in turn, activates a histone chaperone HIRA, and culminates in formation of specialized punctate domains of facultative heterochromatin, called Senescence-Associated Heterochromatin Foci (SAHF), that are enriched in the histone variant, macroH2A. SAHF are thought to repress expression of proliferation-promoting genes, thereby contributing to senescence-associated proliferation arrest. We asked whether this Wnt2-HIRA-SAHF pathway is conserved in mouse fibroblasts.

**Results:**

We show that mouse embryo fibroblasts (MEFs) and mouse skin fibroblasts, do not form robust punctate SAHF in response to an activated Ras oncogene or shortened telomeres. However, senescent MEFs do exhibit elevated levels of macroH2A staining throughout the nucleus as a whole. Consistent with their failure to fully activate the SAHF assembly pathway, the Wnt2-HIRA signaling axis is not overtly regulated between proliferating and senescent mouse cells.

**Conclusions:**

In addition to the previously defined differences between mouse and human cells in the mechanisms and phenotypes associated with senescence, we conclude that senescent mouse and human fibroblasts also differ at the level of chromatin and the signaling pathways used to regulate chromatin. These differences between human and mouse senescence may contribute to the increased propensity of mouse fibroblasts (and perhaps other mouse cell types) to become immortalized and transformed, compared to human cells.

## Background

Cellular senescence is an irreversible proliferation arrest that is an important tumor suppression mechanism and is also thought to contribute to organismal aging [1]. Senescence occurs in response to various cell stresses, including activated oncogenes, critically short telomeres or DNA damage. Senescence as a response to shortened telomeres is termed replicative senescence, and as a response to oncogene activation is termed oncogene-induced senescence. By permanently exiting the cell cycle in the presence of an activated oncogene or exposed telomere ends, the cell is thought to prevent acquisition of additional genetic alterations and possible transformation. In this way, senescence is thought to contribute to tumor suppression. However, senescence may come at a cost to the organism, as this process is also thought to lead to exhaustion of stem cell populations and subsequent tissue and organismal aging.

Comparison of senescence signaling pathways in mouse and human cells has revealed some similarities, but also many differences, between the senescence programs of these two most-studied species [2]. These differences might bear on the different longevity and tumor suppression capacity of these species. Cellular senescence is induced by concerted activity of the p53 and pRB tumor suppressor pathways in most primary human cells, including fibroblasts [3,4]. The pRB protein contributes to cell cycle arrest via its inhibitory effects on E2F family members, transcriptional activators of S-phase genes [5]. The p53 protein also drives many aspects of the senescence program, including cell cycle arrest via transcriptional activation of its downstream effector p21, a cyclin dependent kinase inhibitor [6]. In mouse fibroblasts, inactivation of either the p53 pathway or pRB (together with related proteins, p107 and p130) is generally sufficient to abrogate senescence [7-9]. In contrast, in human fibroblasts, inactivation of both pathways is required to inactivate senescence [3,4,10]. Another important difference between mouse and human cells with respect to senescence induction is expression of telomerase. Because of the 'end-replication problem' and lack of a robust telomere maintenance mechanism, in most proliferating human cells telomeres have potential to become critically short, and thus be sensed as DNA damage and so induce senescence [1]. Mouse somatic cells, however, express telomerase and contain markedly longer telomeres, and thus telomere shortening does not appear to contribute to mouse cell senescence [11-14].

A hallmark of many cultured human cells that have undergone senescence is the formation of regions of characteristically punctate heterochromatin, termed Senescence Associated Heterochromatin Foci (SAHF) [15,16]. The formation of heterochromatin in senescent cells is thought to contribute to permanent growth arrest by silencing proliferating promoting genes, such as cyclin A2, within dense regions of transcriptionally inactive heterochromatin. In human cells, SAHF can be visualized by conventional epifluorescence microscopy as punctate domains of DAPI-stained chromatin. These areas of heterochromatin also have been shown to harbor various well-characterized heterochromatin proteins, such as Heterochromatin Protein 1 (HP1) and histone variant macroH2A.

We have shown previously that in human cells canonical Wnt signaling is an important regulator of SAHF formation [17]. Wnt ligands are extracellular signaling molecules that are important regulators of development, body axis formation and stem cell renewal [18]. Canonical Wnts ligands, when they bind to their cognate transmembrane frizzled receptors, cause disruption of a complex of proteins, including Axin, APC and **G**lycogen **S**ynthase **K**inase 3 (GSK3 (α and β isoforms). In turn, this results in a block to phosphorylation and degradation of a GSK3 substrate, β-catenin, a key transcription effector of canonical Wnt ligands. In pre-senescent primary human fibroblasts, expression of the canonical Wnt ligand, Wnt2, is down-regulated, leading to activation of GSK3β which phosphorylates another of its substrates, the histone chaperone protein, HIRA. As a histone chaperone, HIRA is able to assemble DNA and histones into nucleosomes [19]. When phosphorylated by GSK3β on serine 697, HIRA translocates to specific subnuclear organelles, termed nuclear PML bodies [17]. This translocation of HIRA to PML bodies is a pre-requisite for HIRA to promote formation of punctate SAHF in human cells [20], presumably dependent on its histone chaperone activity. In human cells, formation of SAHF also depends on a functional pRB pathway [15,21]. Hence, in human fibroblasts, the Wnt2-HIRA-SAHF signaling axis and the pRB tumor suppressor pathway coordinately regulate formation of SAHF.

In light of differences between mouse and human fibroblasts in their utilization of the p53 and pRB pathways, and the impact of telomeres on senescence, we hypothesized that there might be differences in the Wnt2-HIRA-SAHF signaling pathway between senescence in mouse and human fibroblasts. Therefore, we sought to determine whether this pathway is also regulated as mouse fibroblasts enter senescence.

## Results

### Senescent mouse fibroblasts do not form robust SAHF

**Figure 1:**
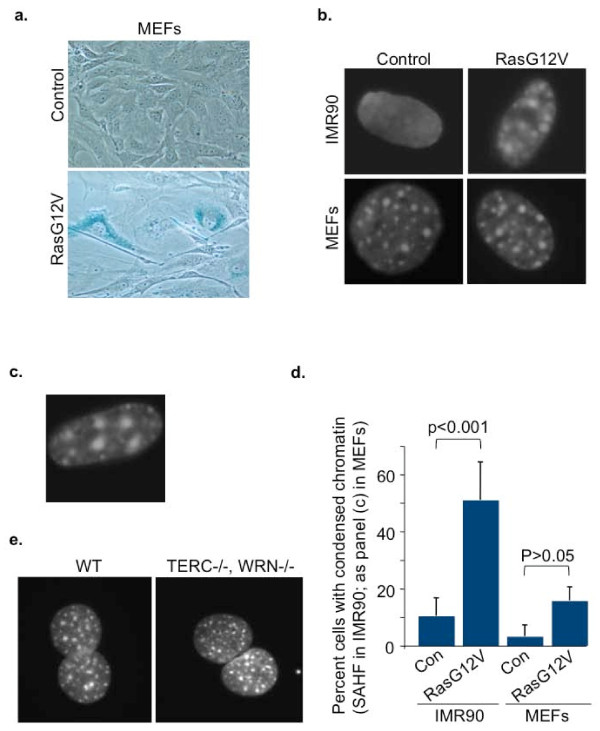
**Lack of marked chromatin changes in senescent mouse fibroblasts**. (a) SA β-gal staining in MEFs transduced with either control or H-RasG12V, drug selected and stained 7 days after drug selection. (b) DAPI staining of nuclei of cells shown in (a) and human IMR90. (c) MEF nucleus with exceptionally condensed heterochromatin. (d) Percent cells with condensed chromatin in senescent MEFs or IMR90 cells. In IMR90, cells with characteristic SAHF as in (b) were scored as positive. In MEFs, only cells with exceptional compaction as in (c) were scored as positive. Pericentromeric heterochromatin of normal MEFs as in (b) was not scored as positive. (e) DAPI staining of wild type mouse skin fibroblasts and generation 5 TERC-/-, WRN-/- skin fibroblasts.

Most cultured primary human fibroblasts, including IMR90, WI38 and BJ strains, exhibit formation of SAHF in response to an activated H-Ras oncogene, typically delivered via a retrovirus ([15] and data not shown). In order to address whether primary mouse fibroblasts similarly form SAHF when senescent, we transduced primary **M**ouse **E**mbryonic **F**ibroblasts (MEFs) with a constitutively active form of H-Ras (H-RasG12V) and assessed changes in chromatin by DAPI staining. As shown previously, in the presence of an activated Ras oncogene, both human fibroblasts (IMR90) and MEFs ceased proliferating, whereas controls transduced with vector alone continued to proliferate (data not shown). The Ras-infected MEFs also acquired a characteristic senescent morphology, typically large, flat, vacuolated and often multi-nucleate, and expressed **S**enescence-**A**ssociated β-galactosidase (SA β-gal) activity (Figure 1a). As expected, Ras-expressing human fibroblasts formed marked SAHF revealed by punctate DAPI-stained domains, which were absent from control-infected cells. However, the vast majority of MEFs exhibited no discernible change in their chromatin in this assay (Figure 1b). Of note, even proliferating MEFs harbor clearly visible domains of bright DAPI-stained constitutive pericentromeric heterochromatin [22,23], and this remained visible in senescent MEFs. Only a small minority of Ras-expressing MEFs appeared to show a marked change in chromatin conformation in this assay, reflected in formation of apparently larger domains of bright DAPI-stained chromatin (Figure 1c-d). Characteristically, these domains of heterochromatin were not only larger than the domains of pericentromeric heterochromatin in proliferating MEFs, but were also fewer in number. Although these cells were apparent by microscopy, the extent of their increase in senescence did not reach significance (Figure 1d). In sum, this result shows that onset of oncogene-induced senescence in most MEFs is not associated with marked changes in chromatin structure, as measured by DAPI staining.

Another trigger of senescence and SAHF formation in human cells is shortened telomeres, associated with replicative senescence [21]. To ask whether shortened telomeres can trigger SAHF formation in mouse cells, we compared wild type mouse skin fibroblasts and skin fibroblasts from generation seven mice lacking both the telomerase catalytic subunit and the Werner's helicase (TERC-/-, WRN-/-). The telomeres of these cells are functionally compromised, leading to marked impairment of cell proliferation and indicators of senescence [24]. However, even when these cells arrested proliferation, we did not observe marked changes in chromatin structure by this assay (Figure 1e). These results indicate that neither activated oncogenes nor shortened telomeres induce changes in chromatin structure in mouse cells that are as marked as those observed in human cells.

**Figure 2:**
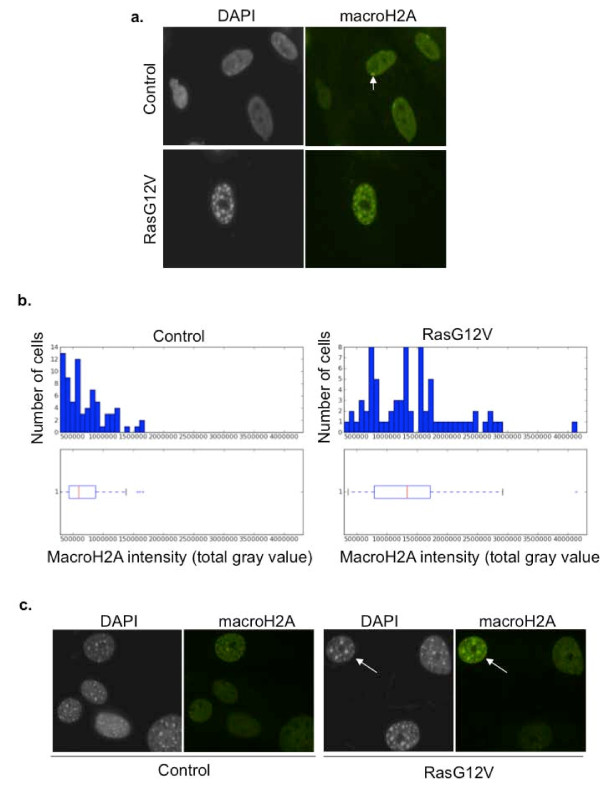
**Senescent MEFs stain more intensely with histone variant macroH2A**. (a) Human IMR90 fibroblasts transduced with either control or H-RasG12V were stained for histone variant macroH2A and DNA (DAPI). Inactive X chromosome is enriched in macroH2A in female cells (arrow). (b) Control or H-RasG12V-transduced senescent MEFs were stained for histone variant macroH2A. Results were quantitated using metamorph and expressed as histograms and box plots: The red line is the median (50th percentile); the box itself encompasses the 25th and 75th percentiles (Inter Quartile Range (IQR)); the whiskers are the most extreme data points within 1.5 × IQR; crosses outside the whiskers are outliers. (c) Images of cells stained as in (b). White arrows indicate a cell with increased macroH2A stain.

In order to investigate further the status of chromatin in senescent MEFs, we examined the incorporation of one component of SAHF, the histone variant macroH2A, by immunofluorescence. MacroH2A is important in heterochromatin mediated silencing of the female X chromosome and as such, in control female-derived human fibroblast IMR90 cells, the barr body stains positively for this protein (Figure 2a) [25]. In addition, as shown previously, macroH2A is incorporated into SAHF in these cells [26]. When we examined MEFs made senescent by transduction of activated Ras (Figure 2b-c), we found that there was an increase in macroH2A staining in many senescent cells, compared to proliferating cells (p < 1 × 10^-11^). Interestingly, this increase in macroH2A staining was most apparent in the small proportion of cells that did show some chromatin compaction in senescent cells (Figure 1c and 2c).

Together, these microscopy-based assays reveal both differences and similarities in chromatin regulation between senescent human fibroblasts and MEFs. A sizeable proportion of the population does show an increase in macroH2A staining throughout the cell nucleus. However, unlike human fibroblasts, the majority of MEFs do not exhibit pronounced changes in chromatin as they become senescent, as judged by formation of DAPI- or macroH2A-stained puncta.

### Senescent mouse fibroblasts do not recruit HIRA to PML bodies

**Figure 3:**
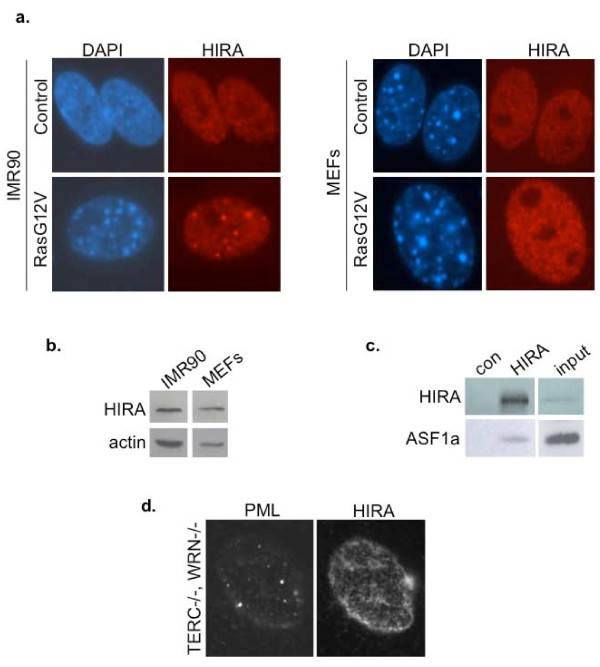
**HIRA is not recruited to PML bodies in senescent MEFs**. (a) Localization of HIRA in control and H-RasG12V transduced IMR90 cells or MEFs was analyzed by immunofluorescence. (b) Expression of HIRA in IMR90 cells and MEFs was verified by western blotting. Actin serves as loading control. (c) Immunoprecipitation-western analysis showing the interaction between HIRA and ASF1a in MEFs. (d) Localization of HIRA and PML in wild type and generation 5 TERC-/-, WRN-/- skin fibroblasts was analyzed by immunofluorescence

Given the established link between HIRA's localization to PML bodies and formation of SAHF in senescent human fibroblasts [26], we questioned whether the failure of senescent mouse cells to form robust punctate SAHF is linked to different regulation of HIRA in these cells. As before, MEFs were made senescent by transduction with an activated Ras oncogene, and senescence confirmed by decreased incoporation of BrdU and positive staining for SA β-gal (data not shown). However, when we compared the subcellular localization of HIRA in proliferating and senescent MEFs and human fibroblasts, we found that HIRA was recruited to PML bodies in senescent human fibroblasts, but not senescent MEFs (Figure 3a and Additional file 1: Figure S1). Of particular significance, HIRA did not localize to PML bodies even in the small proportion of senescent MEFs that did contain compacted heterochromatin, as revealed by DAPI staining (Figure 1c and data not shown). We confirmed that the HIRA protein was expressed in MEFs, and reactive with the same mouse monoclonal antibody used for immunofluorescence (Figure 3b). We also verified that in MEFs, HIRA binds to its partner ASF1a, an interaction shown to be critical for formation of SAHF (Figure 3c) [26]. Similar confirmatory results were also obtained with two rabbit polyclonal antibodies to HIRA (data not shown). We also failed to observe HIRA localized to PML bodies in TERC-/-, WRN-/- skin fibroblasts (Figure 3d). These results indicate that the HIRA chaperone is regulated differently during senescence of mouse and human fibroblasts. Specifically, HIRA is not recruited to PML bodies as MEFs become senescent.

### Senescent MEFs do not downregulate expression of Wnt2

**Figure 4:**
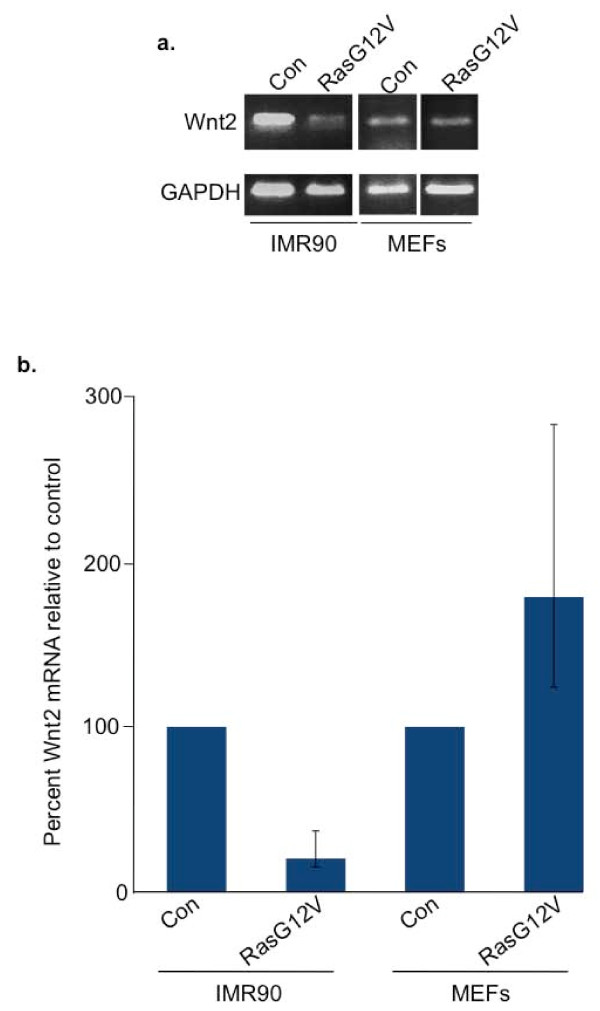
**Senescent MEFs do not downregulate expression of Wnt2**. (a) Semiquantitative RT-PCR analysis of expression of Wnt2 and GAPDH in MEFs and IMR90 cells. (b) Quantitative RT-PCR analysis of expression of Wnt2 and GAPDH in MEFs and IMR90 cells.

In human cells, an early trigger of senescence and recruitment of HIRA to PML bodies is repression of a specific Wnt ligand, Wnt2 [17]. Therefore, we next examined whether MEFs downregulate expression of this specific Wnt ligand. We performed RT-PCR analysis in proliferating and senescent MEFs to determine whether the expression of Wnt2 changed as the cells senesced. Senescence of MEFs was confirmed by appropriate morphological changes and expression of SA β-gal (data not shown). Unlike senescent IMR90 cells, which were compared in parallel as a control, we found that MEFs do not downregulate expression of Wnt2 as they undergo oncogene-induced senescence (Figure 4a-b). This observation extends the differences in regulation of the Wnt2-HIRA-SAHF pathway in MEFs and human fibroblasts.

### Forced activation of GSK3 does not regulate HIRA and SAHF in MEFs

**Figure 5:**
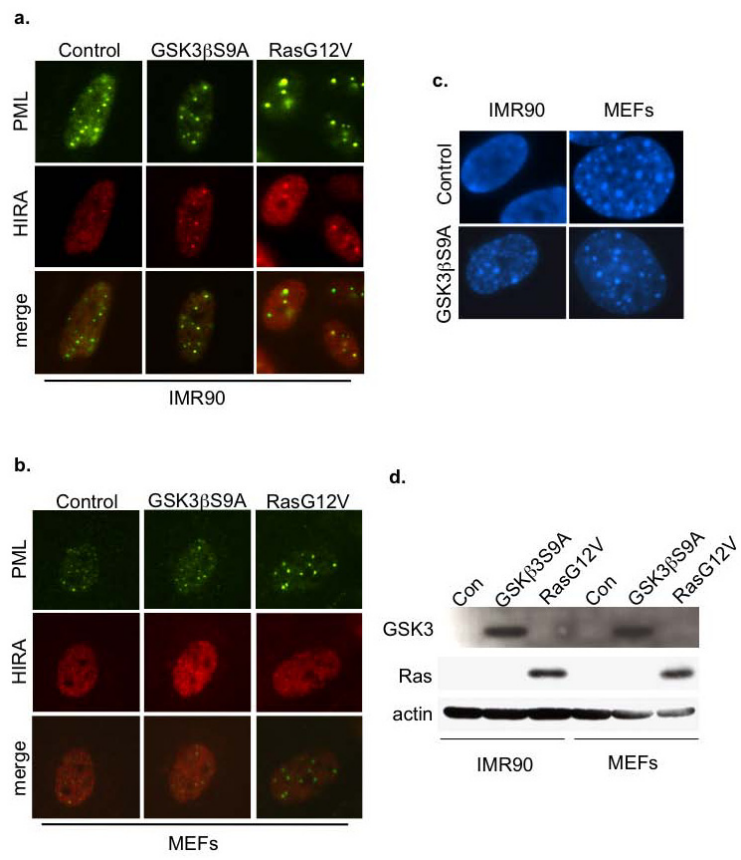
**The HIRA-SAHF pathway is not induced by activated GSK3βS9A in MEFs**. (a) Localization of HIRA and PML in control virus and H-RasG12V-transduced senescent IMR90 cells was examined by immunofluorescence. (b) MEFs examined as in (a). (c) DAPI-staining of representative cells from (a) and (b). (d) Expression of transduced constructs was verified by western blotting V5-tagged GSK3βS9A or Ras.

In human fibroblasts, recruitment of HIRA to PML bodies depends on repression of expression of Wnt2 and consequent activation of GSK3 [17]. Forced activation of GSK3, specifically the GSK3β isoform, has been shown to trigger HIRA's recruitment to PML bodies and formation of SAHF. To test whether GSK3β can similarly induce HIRA relocalization and formation of SAHF in MEFs, we utilized a mutant, GSK3βS9A. In this mutant, the change from serine to alanine at amino acid nine prevents inhibitory phosphorylation of GSK3β [27]. We transduced both human and mouse cells with GSK3βS9A or control vector, and compared HIRA foci and SAHF formation between species. As expected, GSK3βS9A induced HIRA foci and SAHF formation in senescent human cells (Figure 5a). However, this mutant lacked comparable activity in MEFs (Figure 5a-b). We verified that the V5-tagged-GSK3βS9A was expressed in both MEFs and human fibroblasts by western blotting (Figure 5c). These experiments reveal another difference between human fibroblasts and MEFs. Specifically, that forced activation of GSK3β is able to activate the Wnt2-HIRA-SAHF pathway in human fibroblasts, but not MEFs.

## Discussion

In both mice and humans, cellular senescence is thought to contribute to tumor suppression and tissue aging [1]. However, humans and mice show very different lifespans and tumor suppression capabilities. Specifically, humans can live 80 years or more and often suppress the onset of cancer for that long, whereas mice live 2-3 years and can often be afflicted by cancer in that time. That the basis for these profoundly important differences can be at least partly explained at a cellular level, rather than a systemic one, is underscored by the finding that mouse cells are much easier to transform *in vitro *than are human cells [28,29]. Of note, these seminal studies were performed in fibroblasts. In light of this, it is important to describe and understand the differences between the cellular senescence programs of mouse and human fibroblasts. To that end, several differences have already been identified, notably in the relative roles of the pRB pathway and in shortened telomeres.

In this study, we have compared the regulation of the Wnt2-HIRA-SAHF pathway in senescent mouse and human fibroblasts. In doing so, we have uncovered additional differences between these two model systems of senescence. First, mouse fibroblasts differ from human fibroblasts in their ability to form robust punctate SAHF. Specifically, unlike human fibroblasts, the majority of mouse fibroblasts do not exhibit pronounced changes in chromatin as they become senescent, as judged by formation of DAPI- and macroH2A-stained foci. Interestingly, however, a considerable proportion of the population does show increased macroH2A staining, without marked change in organization revealed by DAPI-staining. A small population, which did not reach statistical significance in these studies, did appear to exhibit nuclear reorganization and chromatin compaction, based on DAPI and macroH2A staining. Second, senescent mouse fibroblasts do not recruit the histone chaperone HIRA to PML bodies. Third, MEFs that have been induced to senesce in response to activated Ras do not downregulate expression of Wnt2. Fourth, while ectopic expression of uninhibitable GSK3β in human fibroblasts is sufficient to trigger recruitment of HIRA to PML bodies and formation of SAHF in human cells, it fails to do so when ectopically expressed in MEFs. Together, these results indicate that, compared to human fibroblasts, mouse fibroblasts are markedly impaired in their ability to activate and signal through the Wnt2-HIRA-SAHF pathway.

More specifically, these results point to at least two differences between human fibroblasts and mouse fibroblasts in this Wnt2-HIRA-SAHF pathway. First, senescence of mouse fibroblasts is not associated with the trigger that is responsible for activation of this pathway in human cells, namely repression of Wnt2. Second, mouse fibroblasts and human fibroblasts also differ downstream of Wnt2, as indicated by the ability of GSK3βS9A to induce SAHF in human, but not mouse, fibroblasts. Why senescent mouse fibroblasts fail to repress expression of Wnt2, and the difference between mouse and human fibroblasts downstream of Wnt2, are issues that remain to be resolved. Regardless of the basis for these differences, it will ultimately be important to determine whether the relative ease of transformation of mouse cells, compared to human cells [28,29], stems, at least in part, from their failure to regulate this Wnt2-HIRA-SAHF axis, a candidate tumor suppressor pathway.

The mechanism by which HIRA's recruitment to PML bodies contributes to formation of SAHF is not well understood. Previous studies have indicated that HIRA's relocation to this subnuclear organelle is required for formation of SAHF, because blocking its relocalization, either with a dominant negative HIRA mutant or with the PML-RARα fusion protein which disrupts PML bodies, abolishes formation of SAHF [20,21]. This study further emphasizes the link between HIRA's localization to PML bodies and formation of SAHF, because the failure of mouse fibroblasts to obviously form punctate SAHF correlates with a failure of HIRA to enter PML bodies in these cells. Conversely, these results imply that recruitment of HIRA to PML bodies is not directly linked to regulation of macroH2A. This is consistent with HIRA being primarily involved in deposition of histone (H3/H4)2 heterotetramers, rather than H2A/H2B heterodimers [19].

In this study, we note a level of similarity between the appearance of DAPI-stained nuclei of proliferating non-senescent MEFs and senescent human cells. This similarity at the microscopic level is not founded at the molecular level. Specifically, the bright-stained DAPI puncta in mouse fibroblasts reflect the DNA sequence and structure of mouse pericentromeric heterochromatin [22,23]. However, the SAHF of senescent human cells largely exclude pericentromeres and telomeres [20,21]. The similarity at the microscopic level is potentially confusing, and we caution that a punctate DAPI stain should not be used to score senescence of MEFs, as it sometimes is in human cells.

## Conclusions

Previous works of others have found important functional differences between the senescence programs of mouse and human fibroblasts. As detailed in the Background, there are important differences in the role of the pRB and p53 tumor suppressor pathways and telomeres. The p53 pathway is dominant in mouse fibroblasts, whereas in human cells either activation of the p53 or pRB pathway leads to senescence. To these key differences between senescence of mouse and human fibroblasts, we add differences in the regulation and function of the Wnt2-HIRA-SAHF pathway. Based on several measures, this pathway is not overtly regulated at the onset of senescence in MEFs, compared to its regulation in human fibroblasts. However, our results obviously do not exclude some role for HIRA in regulation of senescence in MEFs, and future more detailed studies will be required to fully define the similarities and differences in chromatin regulation between mouse and human cells.

## Methods

### Cell Culture

IMR90 (ATCC) cell lines were cultured according to ATCC guidelines in ambient oxygen. Experiments were performed on IMR90 cells between population doubling (PD) 20 and PD 35. Mouse Embryo Fibroblasts (MEFs) were prepared from pooled wild type C57BL/6J embryos that were gestational age E12-E14. The head and internal organs were removed, and the torso was minced and dispersed in 0.25% trypsin + EDTA for 15 minutes in shaking water bath at 37°C. Cells were spun, debris removed and then plated. These cells were considered passage 0. MEFs used in experiments were cultured in ambient oxygen and experiments were performed between PD 1 and PD 8. MEFs were cultured in Dulbecco's modified Eagle's medium (DMEM) supplemented with 10% fetal bovine serum. Cells were harvested 7 days post drug selection for all assays. Mouse adult ear skin fibroblasts were obtained as described [24].

### DNA constructs and retroviral transduction

The following plasmids were used to produce retroviruses: pBabe-puro-H-RasG12V (a gift of Bill Hahn and Bob Weinberg); pBabe-puro-GSK3βS9A from Addgene (plasmid #14128). Retroviral-mediated gene transfer was performed using the Phoenix packaging cells (Dr. Gary Nolan, Stanford University). Phoenix cells were transfected using Polyethylenimine (PEI), Linear MW 25,000 (Polysciences, Inc. Warrington, PA) sterile solution 1 mg/ml. To one milliliter of serum free DMEM, 16 micrograms of retroviral plasmid DNA and 2 micrograms of a plasmid encoding vesicular stomatitis virus glycoprotein G (VSV-G) plus 38 micrograms of PEI was added, this solution was briefly vortexed, left to sit for 10 minutes and then added to Phoenix Cells. Twenty-four hours later, media was changed on Phoenix cells. At 48 hours post transfection, virus-containing medium was collected, supplemented with 8 μg/ml of polybrene (Sigma), and incubated with the target IMR90 or MEF cells at 37°C for 24 hours. A second round of infection was performed on the same target cells. MEFs and IMR90 cells were transduced in parallel and drug selected with 3 μg/ml or 1 μg/ml puromycin, respectively.

### Immunofluorescence, antibodies, SAHF, and SA β-gal staining

Two color indirect immunofluorescence assays were performed as described previously [26,30]. Anti-PML (AB1370, Chemicon) Anti-PML for Additional file 1: Figure S1 (Upstate 05-718) Anti-V5 (Invitrogen) Anti-Ras (BD Transduction Labatories Cat. No. 610001), Anti-beta actin (Abcam clone AC-15) were from the indicated suppliers. Anti-macroH2A and anti-HIRA antibodies were described previously [20,31]. SAHF (DAPI foci) were detected by staining with 0.13 μg/ml DAPI for 2 min at room temperature (as opposed to standard conditions of 1 μg/ml for 5 min). SA beta-gal staining was performed as described previously [32].

### Coimmunoprecipitations

To prepare cell lysates, cells were washed once with PBS and then collected by incubating in EBC-500 (50 mM Tris-HCl pH8, 500 mM NaCl, 0.5% NP-40 + protease inhibitors) for 20 minutes while rocking at 4°C, scraped into microcentrifuge tubes, passed through a 22 gauge needle five times and then spun for 10 minutes at 10,000 × g. Supernatant was used for immunoprecipitations. Immunoprecipitation was performed using 2 mg of extracted protein for 3 hours at 4°C. Antibodies used for IP were WC15 [31] and M7023 secondary (Sigma, St. Louis, MO). Immunoprecipitates were recovered after 1 hour incubation at 4°C with protein A Sepharose Beads (Amersham/GE healthcare, Baie d'Urfé, QC, Canada). Precipitates were washed five times with NETN (20 mM Tris pH8, 1 mM EDTA, 0.5%NP-40, 100 mM NaCl). Precipitates were eluted in 50 ul of 1 × Laemmli sample buffer, boiled 5 minutes, and separated by SDS-PAGE and transferred to Immun-Blot PVDF membrane (Biorad, Hercules, CA).

### RT-PCR

Total RNA was prepared using Trizol (Invitrogen), according to the manufacturer's instructions. Reverse transcription-PCR (RT-PCR) was performed using the Qiagen one-step RT-PCR kit, according to the manufacturer's instructions. Primers used for human GapDH GAGAGACCCTCACTGCTG and GATGGTACATGACAAGGTGC and for human Wnt2 ATGAACGCCCCTCTCGGTGGAA and TGTCCTTGGCGCTTCCCATC. For Mouse Wnt2: 5'-GTACATGAGAGCTACAGGTGG-3' and 5'-ACGGGCAAACTTGATCCCGTAGTC-3' and GAPDH: 5'-GATGATGACCCTTTTGGCTCCACC-3' and 5'-CCACTCACGGCAAATTCAACGGCA-3'. Quantitative RT-PCR was performed using the one-step RT-PCR Master mix (Applied Biosystems). Real time PCR and cDNA synthesis was performed using the following mixture: 25 μl 2 × reaction buffer, 1.25 μl 40 × multiscribe, 2.5 μl 20 × primer/probe and 100 ng RNA in a 50 μl total volume. Reactions were amplified and analyzed in triplicate using Chromo-4 real-time PCR detection system (Bio-rad). The following primer/probe sets (Applied Biosystems) were used: human wnt2 Hs00608222, human beta-actin (endogenous control-4352935E), mouse wnt2 Mm00470018, mouse beta-actin Mm01205647.

## Competing interests

The authors declare that they have no competing interests.

## Authors' contributions

ALK performed all the experiments and wrote the manuscript. TM performed statistical analysis of data. GHE participated in design of the study and provided funding for ALK. FBJ participated in design of the study. RZ participated in design of the study. PDA conceived the study, participated in the design, provided funding and wrote the manuscript.

All authors read and approved the final manuscript.

## Supplementary Material

Additional file 1 **Figure S1: HIRA is not recruited to PML bodies in senescent MEFs**. Localization of HIRA and PML in H-RasG12V transduced MEFs was analyzed by immunofluorescence. Click here for file
