# Supplementary figures and images for: Senescent mouse cells fail to overtly regulate the HIRA histone chaperone and do not form robust Senescence Associated Heterochromatin Foci
Source: Cell Div. 2010 Jun 22;5:16. doi: 10.1186/1747-1028-5-16 (PMC2904742; doi:10.1186/1747-1028-5-16)

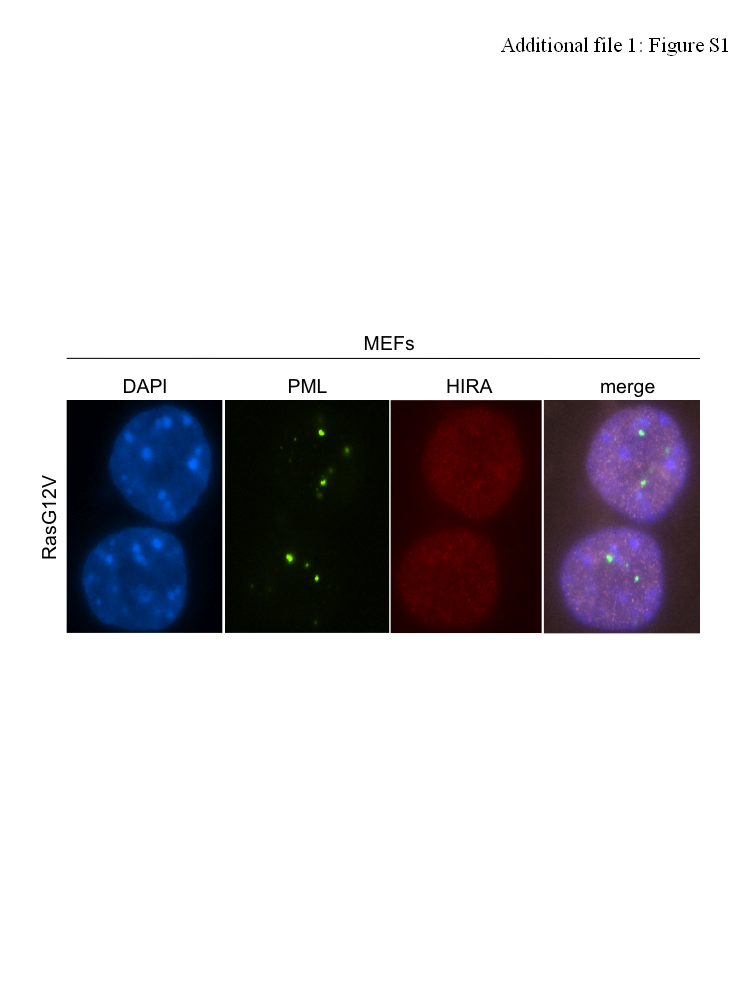

Supplement: Additional file 1 — Figure S1: HIRA is not recruited to PML bodies in senescent MEFs. Localization of HIRA and PML in H-RasG12V transduced MEFs was analyzed by immunofluorescence. [file 1747-1028-5-16-S1.TIFF]
